# Prestin kinetics and corresponding frequency dependence augment during early development of the outer hair cell within the mouse organ of Corti

**DOI:** 10.1038/s41598-019-52965-1

**Published:** 2019-11-11

**Authors:** Jun-Ping Bai, Dhasakumar Navaratnam, Joseph Santos-Sacchi

**Affiliations:** 1 Department of Surgery (Otolaryngology), Yale University School of Medicine, 333 Cedar St, New Haven CT, USA; 2 Department of Cellular and Molecular Physiology, Yale University School of Medicine, 333 Cedar St, New Haven CT, USA; 3 Department of Neuroscience, Yale University School of Medicine, 333 Cedar St, New Haven CT, USA; 4 Department of Neurology, Yale University School of Medicine, 333 Cedar St, New Haven CT, USA

**Keywords:** Kinetics, Membrane biophysics

## Abstract

Several studies have documented the early development of OHC electromechanical behavior. The mechanical response (electromotility, eM) and its electrical correlate (nonlinear capacitance, NLC), resulting from prestin’s voltage-sensor charge movement, increase over the course of several postnatal days in altricial animals. They increase until about p18, near the time of peripheral auditory maturity. The correspondence of auditory capabilities and prestin function indicates that mature activity of prestin occurs at this time. One of the major requirements of eM is its responsiveness across auditory frequencies. Here we evaluate the frequency response of prestin charge movement in mice over the course of development up to 8 months. We find that in apical turn OHCs prestin’s frequency response increases during postnatal development and stabilizes when mature hearing is established. The low frequency component of NLC, within *in situ* explants, agrees with previously reported results on isolated cells. If prestin activity is independent of cochlear place, as might be expected, then these observations suggest that prestin activity somehow influences cochlear amplification at high frequencies in spite of its low pass behavior.

## Introduction

The outer hair cell (OHC) enhances cochlear sensitivity up to 3 orders of magnitude, and the cellular basis of this feat resides within the OHCs that populate the organ of Corti. OHCs are electro-motile, responding to transmembrane current perturbations with changes in cell length, termed electromotility (eM)^1,2^. Though the pertinent electromechanical characteristics (e.g., voltage-dependence, and magnitude) of OHC eM had been studied since the mid-80s^3,4^, the identification of the membrane protein, SLC26a5 (prestin), driving this response required over a decade^5^. Various candidates were considered along the way, similar to the quest for the stereociliar MET channel^6^.

Prestin drives OHC electromotility (eM), known to be responsible for cochlear amplification (CA) in mammals^7^. The electrical signature of eM is a bell-shaped nonlinear capacitance (NLC), the first derivative of prestin sensor charge vs. membrane voltage (dQ_p_/dV_m_), which peaks at a characteristic membrane voltage (V_h_)^8,9^. We have previously studied the development of NLC in OHCs of the mouse^10^. Those studies demonstrated that increases in prestin charge (Q_max_) continued after stabilization of linear capacitance (at p10), which corresponds to total membrane surface area (sum of membrane and embedded prestin surface area). Thus, though the number of prestin molecules appeared to stabilize, additional changes in NLC characteristics ensued, indicating some sort of maturational events. Here we study, in developing OHCs, the maturation of prestin kinetics, which has recently been shown to possess low pass characteristics in adult guinea pig OHCs^11^. We find that during development the frequency response of NLC increases, stabilizing near 6 kHz in adults at the apical turn of the cochlea at room temperature. We also find that linear capacitance decreases with aging, and is indicative of reduced prestin insertion in the membrane. Nevertheless, specific motor charge density (a metric for prestin density within the membrane) remains fairly constant.

## Methods

C57/B6 pups aged postnatal days 6–18, and months 2 and 8 were used. All experimental protocols were approved by the Yale Animal Care and Use Committee, and were in accordance with relevant guidelines and regulations. Apical turns of the organ of Corti were dissected out and recorded with ionic current blocking solutions, thereby removing interference on measures of membrane capacitance. The extracellular solution contained (in mM): 100 NaCl, 20 tetraethylammonium (TEA)-Cl, 20 CsCl, 2 CoCl_2_, 1 MgCl_2_, 1 CaCl_2_, 10 HEPES, pH 7.2. The intracellular solution contained (in mM): 140 CsCl, 2 MgCl_2_, 10 HEPES, and 10 EGTA, pH 7.2. Pipettes were coated with M-coat to reduce stray capacitance, and had resistances of 3-5 MΩ. Gigohm seals were made and stray capacitance was balanced out with amplifier circuitry prior to establishing whole-cell conditions. A Nikon Eclipse E600-FN microscope with 40× water immersion lens was used to observe cells during voltage clamp. Whole cell voltage clamp recordings were performed from all rows of outer hair cells (OHCs) of whole mount organ of Corti. We limited the recording area within 1/4 turn. OHCs were recorded at room temperature using jClamp software and an Axopatch 200B amplifier. Data were low pass filtered at 10 kHz and digitized at 100 kHz with a Digidata 1320 A.

Chirp voltage stimuli were delivered across frequency and analyzed within 300–7000 Hz, where stray capacitance was removed. The voltage chirps were generated in jClamp using the Matlab logarithmic “chirp” function (10 mV pk; pts = 4096; F0 = 24.4141 Hz; F1 = 50 kHz; t1 = 0.04095 s). Chirp responses were not averaged at each voltage step (−160 to +120 in 40 mV steps). Cell currents were either averaged for analysis (6–8 cells/group) for surface plot presentations or analyzed individually for statistical measures. Detailed analysis of C_m_ was performed in Matlab. Capacitance was measured using dual-sine analysis at harmonic frequencies (Santos-Sacchi *et al*., 1998; Santos-Sacchi, 2004). Briefly, real and imaginary components of membrane current at harmonic frequencies were determined by FFT in jClamp, corrected for the roll-off of recording system admittance^12^ and residual stray capacitance^13^. R_s_, R_m_ and C_m_ were extracted using the dual-sine, 3-parameter solution of the standard patch clamp model^14,15^, based on the original single sine solution^16^. In order to extract Boltzmann parameters, capacitance-voltage data were fit to the first derivative of a two-state Boltzmann function.

1 $${C}_{m}=NLC+{C}_{sa}+{C}_{{\rm{lin}}}={Q}_{{\rm{\max }}}\frac{{\rm{ze}}}{{k}_{B}T}\frac{b}{{(1+b)}^{2}}+{C}_{sa}+{C}_{{\rm{lin}}}$$

$$\mathrm{where}\,b=exp(-ze\frac{{V}_{m}-{V}_{h}}{{k}_{B}T}),\,{C}_{sa}=\frac{\Delta {C}_{sa}}{(1+{b}^{-1})}$$

Q_max_ is the maximum nonlinear charge moved, V_h_ is voltage at peak capacitance or equivalently, at half-maximum charge transfer, V_m_ is R_s_-corrected membrane potential, *z* is valence, C_lin_ is linear membrane capacitance, e is electron charge, *k*_*B*_ is Boltzmann’s constant, and T is absolute temperature. C_sa_ is a component of capacitance that characterizes sigmoidal changes in specific membrane capacitance^17^. ΔC_sa_ is the total sum of unitary changes per prestin motor protein. Q_sp_ denotes charge density, namely Q_max_/C_lin_.

A Lorentzian function (or the sum of two Lorentzians) (Gale and Ashmore, 1997; Santos-Sacchi and Tan, 2018) was used to fit NLC across frequency.

2 $$NLC(f)=NL{C}_{0}/{[1+{(2\pi f\tau )}^{2}]}^{1/2}$$

where NLC_0_ is the zero frequency component, and τ = 1/F_c_, the cut-off frequency. Additionally, a power fit of NLC across frequency was performed.

3 $$NLC(f)=NL{C}_{0}+a\,\ast \,{f}^{b}$$

where NLC_0_ is the zero frequency component, and *a* and *b* control the frequency response.

## Results

**Figure 1:**
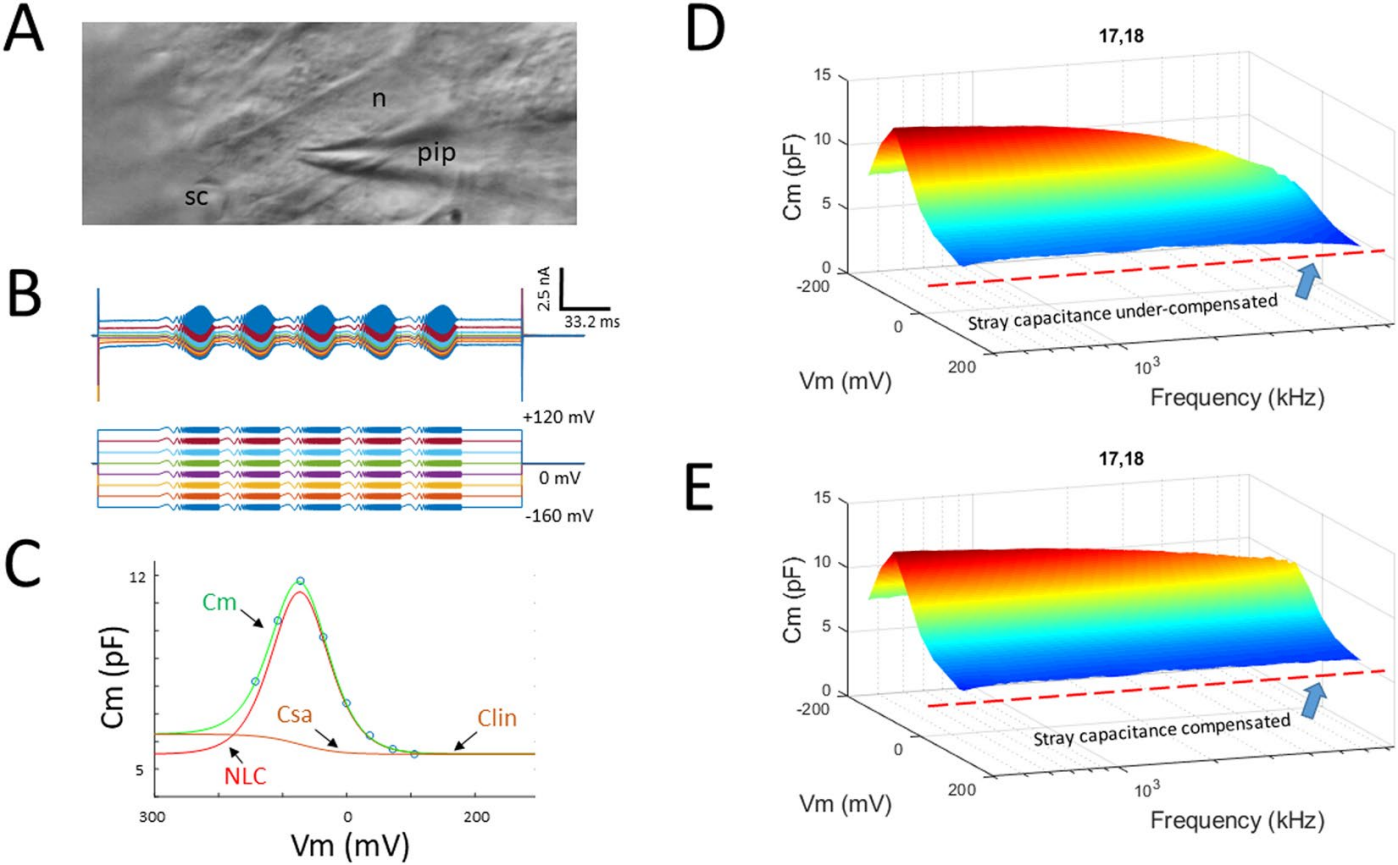
High frequency measurement of OHC NLC in organ of Corti explants. (**A**) Light micrograph of an *in situ* patch clamped OHC. sc, stereocilia; n, nucleus; pip, patch pipette. (**B**) Whole cell currents induced by chirp voltage protocol. (**C**) Derived voltage-dependent capacitance (circles) at each frequency (see methods) was fit to extract NLC and its Boltzmann parameters. (**D,E**) Surface plots of raw C_m_ data before (**D**) and after (**E**) correction for residual stray capacitance effects on high frequency measures. Note frequency independence of C_lin_ following correction.

Figure 1 illustrates our methodology to measure NLC across frequency. With our Boltzmann fit (Eq. 1) we obtain the usual Boltzmann parameters while extracting NLC, and additionally determine C_sa_, a component of capacitance that we believe arises from changes in membrane surface area or membrane thickness as prestin alters its conformational state across voltage^17–20^.

**Figure 2:**
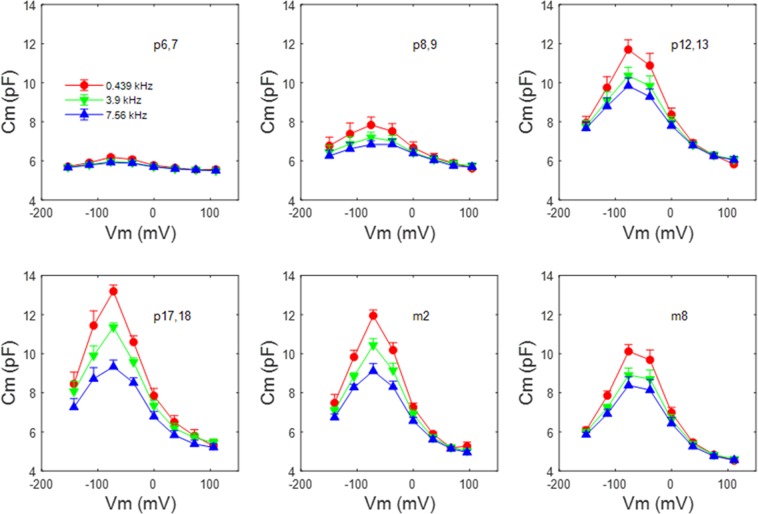
NLC (mean+/− se) as a function of postnatal age at select stimulating frequencies. Peak NLC reaches maximal values at p17/18 across frequency. C_lin_ increases until p12/13 as a result of surface area increase, but decreases thereafter. NLC also reduces following p17/18. By 8 months, C_lin_ has markedly decreased. P6,7 n = 7; p8,9 n = 7; p12,13 n = 6; p17,18 n = 8; 2 month n = 8; 8 month n = 9.

Figure 2 shows mean (+/− se) NLC at 3 selected frequencies within our measurement bandwidth, as a function of postnatal age. As determined previously, NLC reaches maximal values at p17/18 in rodents^10,21^. Here we find the same pattern across frequencies. C_lin_ increases until p12/13 as a result of surface area increase as the cells mature, lengthen and express increasing amounts of prestin within the membrane. However, C_lin_ decreases thereafter at all frequencies. By 8 months, C_lin_ has decreased by more than 25%. NLC also reduces following p17/18. The plots also highlight a substantial roll-off of peak NLC across frequency, which we investigate in more detail further below.

Because we limited our recordings to a quarter turn of the apical coil of the cochlea, we determined whether the variability in hair cell length within that region could impact our C_lin_ data. OHC lengths in different rows of the cochlear apex are indistinguishable^22^. In the mouse strain we used, they determined that OHC lengths from middle (17.3 μm) and apical (21.3 μm) turn regions differed by 4 μm. Assuming that this progression will translate into our quarter turn (as their plots suggest) we would maximally expect about a 1 μm length variability in our hair cell recordings. Given the cylindrical geometry of the OHC and its diameter in mice (~7 μm), we calculate an expected maximal variability in linear capacitance (based on surface area arising from our measured membrane specific capacitance of 0.08 pF/μm^2^^10^ of 0.26 pF. This is well below our finding of −1.6 pF (25% reduction from p12/13) during aging. Utilizing a more stringent estimate of 0.05 pF/μm^2^ for specific membrane capacitance^23^, giving 0.165 pF variability, our results are even less likely to be influenced by sampling within the quarter turn. We conclude that the C_lin_ changes during aging are real.

**Figure 3:**
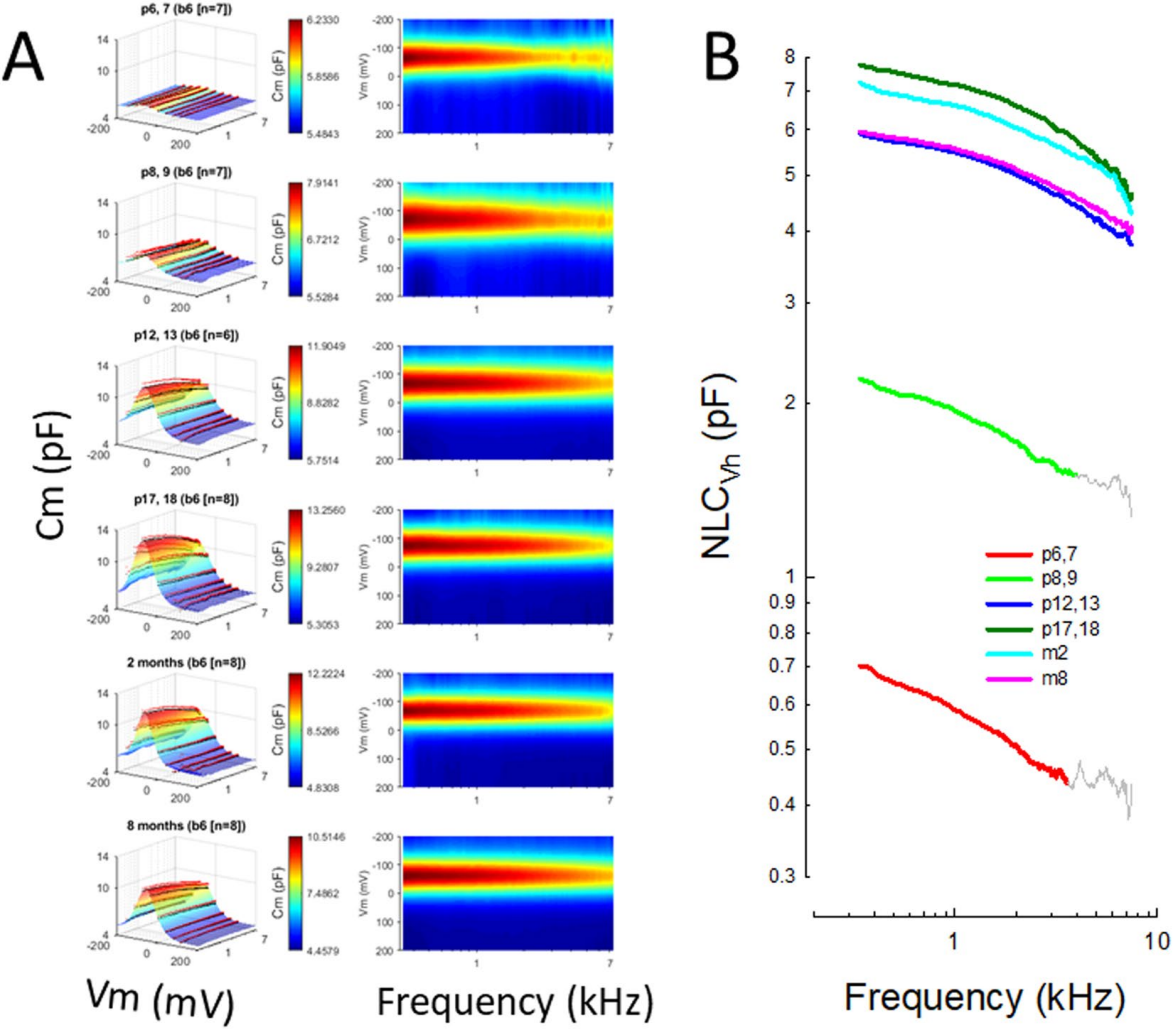
(**A**) Left panels show full NLC spectrum (0.39–7 kHz) surface plots as a function of postnatal age, illustrating the bell-shaped NLC and its roll-off with increasing frequency. Black dots indicate means and pink dots are +se, which sit atop the Boltzmann fits. The panels on the right show the same data top-down, and provide a clearer view of the frequency response changes during aging. (**B**) NLC_Vh_ for each age group is log-log plotted to more clearly show the low-pass behavior that changes during aging. The colored points were used for fitting in Fig. 5. For the 2 youngest groups grey lines show regions omitted from fits because of noise at high frequencies.

In Fig. 3 we show the NLC recorded between 0.39 and 7 kHz at 24 Hz resolution. The data show that NLC is maximal at low frequencies and decreases at higher frequencies, depending on the age of the animal. In Fig. 3A, the 3D plots on the left highlight the voltage dependence of NLC (mean +/− se of the data overlie the smooth Boltzmann fits), whereas those 2D plots on the right highlight the frequency response of NLC. In Fig. 3B, we plot NLC at V_h_, illustrating the changing frequency dependence as animals age. The low pass nature of NLC at all ages is apparent. A quantification of this roll-off is supplied below. Nonetheless, the representations in Fig. 3 provide a rich picture of the magnitude, voltage-dependence and frequency extent of NLC across age.

**Figure 4:**
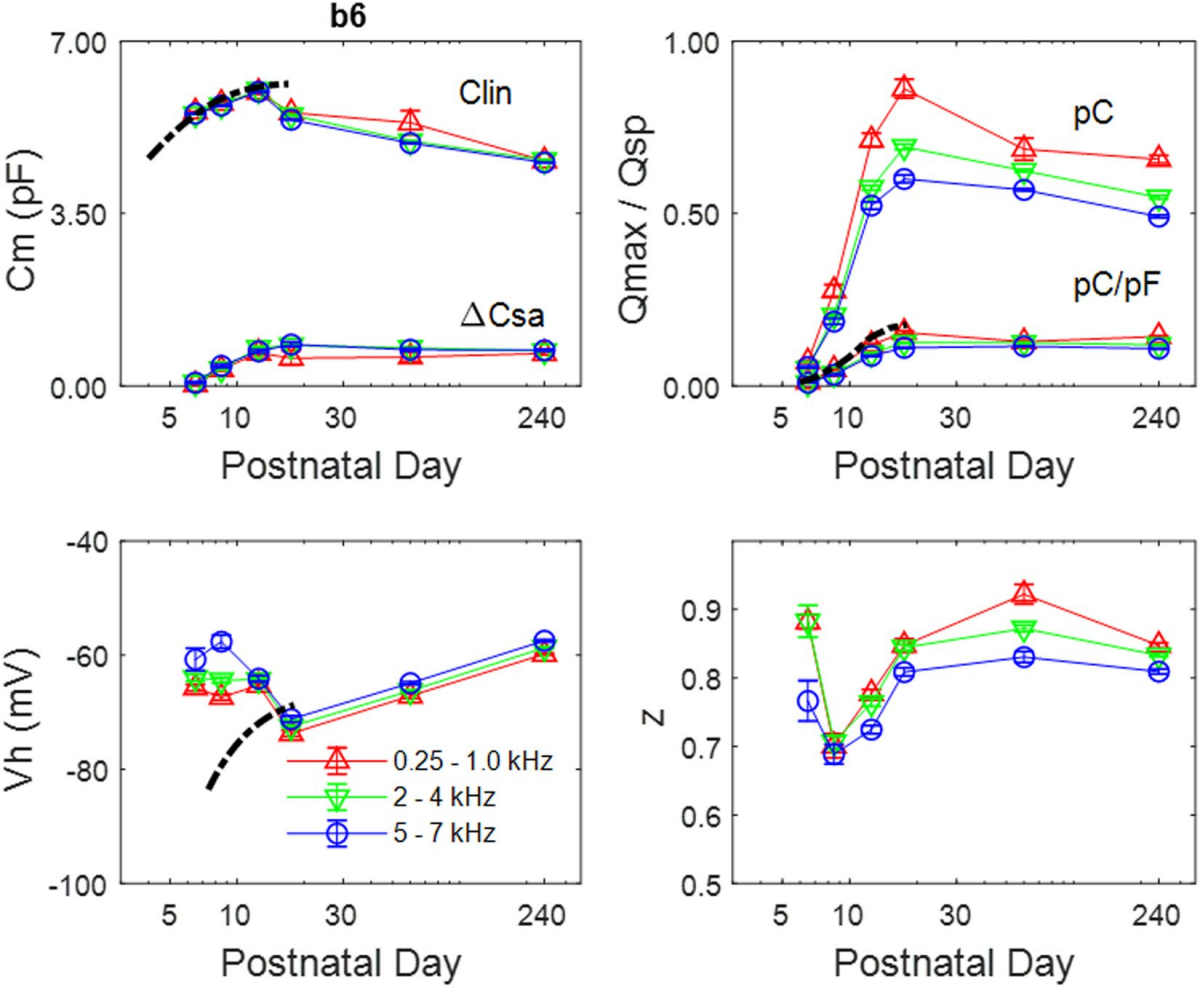
Boltzmann characteristics (mean +/− se) for selected bandwidths. The dashed line denotes NLC measured at low frequencies from isolated OHCs (Abe *et al*., 2010). Note abrupt decline of C_lin_, while C_sa_ remains steady. V_h_ is initially at a more positive potential than isolated cells. Q_max_ (pC) and Q_sp_ (pC/pF) increase rapidly during development. Q_max_ decreases after p17/18, with a clear difference between low and high frequencies, but Q_sp_ stabilizes because of the C_lin_ drop. Following some variability at p6/7, *z* increases with age, and also tends to be smaller at higher frequencies. P6,7 n = 7; p8,9 n = 7; p12,13 n = 6; p17,18 n = 8; 2 month n = 8; 8 month n = 9.

Boltzmann parameters (mean +/− se) for three disparate bandwidths are plotted in Fig. 4 as a function of age. A comparison to our previous isolated cell mouse data^10^ obtained at low frequency (dotted lines) is made. Quantification of Q_max_ and C_lin_ confirms their initial rise between p6/7 to p12/13 as the cells increase in surface area, and mirror measures made in isolated cells. The development of Q_sp_ in the low frequency region also corresponds to measures from isolated cells. After p17/18, Q_max_ decreases, while Q_sp_ appears to stabilize, due to simultaneous decrease in C_lin_. The additional frequency information provided by chirp analysis shows that both Q_max_ and Q_sp_ following p17/18 depend on frequency, with higher frequencies showing reduced values. Interestingly, V_h_ within the explant differs from isolated cells during early development, and may result from forces that normally exist in the organ of Corti, e.g., membrane tension. It is well known that many factors can influence V_h_, including membrane tension and intracellular Cl^−^,^14,24–30^. The Boltzmann parameter *z*, which characterizes the voltage sensitivity of prestin or the distance sensor charge is moved within the membrane field, is variable in p6/7 animals, but clearly increases after this time period over age. Some frequency dependence of *z* in the adult is evident, and reduction in *z* at high frequencies in the adult guinea pig was originally noted by Gale and Ashmore^31^.

**Figure 5:**
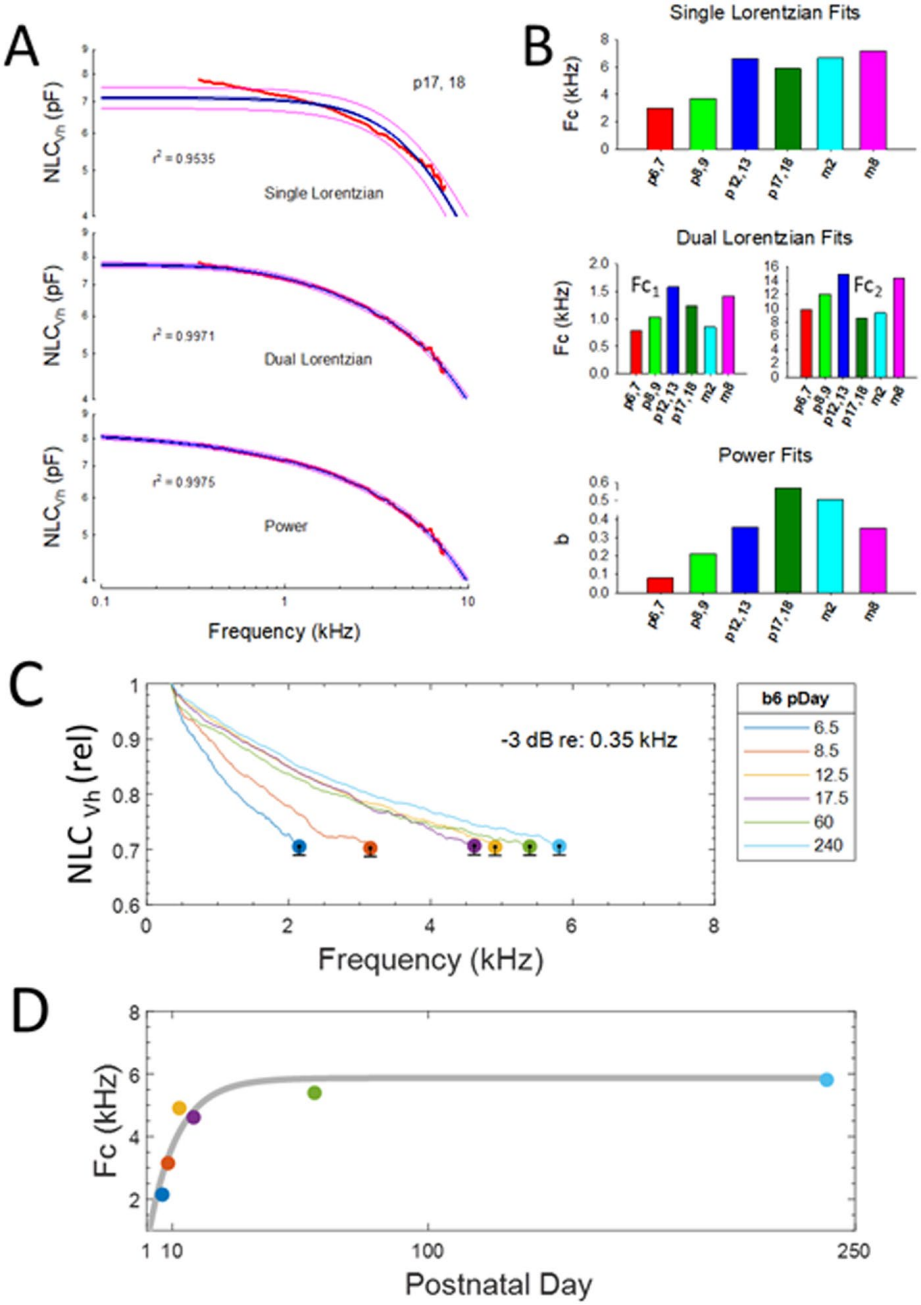
Changes in frequency response of NLC during aging. (**A**) Three types of fits to mean NLC_Vh_ were performed to estimate frequency roll-off. Examples of the 3 fits for p17/18 OHCs are shown. Blue line is fit and red lines are 95% confidence predictions of the fits (done in Sigmaplot). The poorest fit is with a single Lorentzian, followed by dual Lorentzian and power fits (*f* in kHz). Nevertheless, each provides evidence for increases in frequency responsiveness during development. The <a> parameter, as all others, was not constrained, but was not age –dependent and similar for all fits [p6,7 - m8: −1.4055, −1.3548, −1.5980, −1.1960, −1.1552, −1.5031; mean +/− se −1.369 (0.07)]. The small se indicates little variability. (**B**) Bar plots of frequency cut-off parameters of the fits. (**C**) Another metric of frequency response roll-off was to determine the −3dB magnitude of NLC_Vh_ relative to 350 Hz values, denoted here with circles. It should be noted that the Fc’s simply reflect the relative roll-off during aging, and their absolute values will differ depending on the reference frequency. During the life span, Fc increases. The se indicates the variability at the cut-off frequencies. (**D)** The Fc data were fit to a power law function in Matlab (grey line; Fc = a.*(1-b.^pDay), where a = 5.867 and b = 0.9065; R^2^ = 0.849), and indicates a stabilization near 6 kHz. P6,7 n = 7; p8,9 n = 7; p12,13 n = 6; p17,18 n = 8; 2 month n = 8; 8 month n = 9.

Finally, in Fig. 5 we explore the changes in frequency cut-off (Fc) of NLC at V_h_. Since the reduction of NLC_Vh_ across frequency is clearly not a simple process, three methods of fitting were evaluated to estimate frequency roll-off (Fig. 5A). We provide fits to average data. Examples of the 3 fit types for p17/18 OHCs are shown, including single Lorentzian, dual Lorentzian (sum of 2 Lorentzians) and power fits. The blue line depicts the fit and red lines show 95% confidence predictions of the fits (fitting performed in Sigmaplot). The poorest fit is with a single Lorentzian, followed by dual Lorentzian and power fits. In regard to the power fit, we have previously found evidence of multi/stretched exponential behavior in NLC^32,33^. The stretched exponential (known as the Kohlrausch-Williams-Watts [KWW] relaxation function), first devised by Kohlrausch (1854) to describe the behavior of capacitor discharge in a Leyden jar, is also applicable to the viscoelastic behavior of glasses^34^, and other systems where time-dependent behavior results from overcoming a multitude of energy barriers. Interestingly, cytoskeletal dynamics is one such system^35^. The KWW function has a related counterpart in the frequency domain^36^, termed the Havriliak-Negami [HN] relaxation function. Fitting our data with that counterpart, we obtain similar quality fits to the power functions (for example, at p17,18 the fit R^2^ is 0.9975 and 0.9974 for power and HN). Since the power function fit has fewer parameters (one less) than HN, we have chosen to illustrate power fits. The usage of the term *low-pass* for the power function should not be construed in a strict engineering sense, but here denotes domination of low pass components whose slope changes over maturation. Figure 5B shows that regardless of the utilized fitting function, each provides evidence for increases in frequency responsiveness during development, as indicated by the bar plots. The dual Lorentzian gives dubious Fc_2_ cut-offs since they are above the sampled frequency. In Fig. 5C, we provide another metric of frequency response roll-off by finding the −3dB magnitude of NLC_Vh_ relative to 350 Hz. During the life span, Fc increases and appears to stabilize near 6 kHz in our older animals. Interestingly, the cut-offs roughly correspond to those of the single Lorentzian fits. We are cognizant that these cut-off estimates cannot be considered true cut-off frequencies, but only indicate that the NLC response has lost much of its steady state magnitude even at low frequencies. That is, the Fc’s simply reflect the relative roll-off among aging groups, and their absolute values will differ depending on the reference frequency. Thus, all methods to quantify roll-off indicate that frequency responsiveness increases with age. Possible reasons for this are discussed below.

## Discussion

Studying the development of OHC function can reveal important determinants of adult auditory function. A variety of OHC characteristics mature during the course of post-natal development in altricial animals, including stereociliar transduction and eM. By using such animals, these characteristics can be conveniently studied in isolated cells or explants following birth. The development of eM and its corresponding electrical correlate, nonlinear membrane capacitance (NLC), have been found to reach maturity near p18 in the rodent^10,21,37^, close to the onset of hearing. These studies did not include an assessment of prestin frequency dependence during development, a likely important factor for OHC performance in the adult animal’s acoustic environment. Here we evaluated the development of NLC frequency response in the *in situ* mouse cochlea, and find that its frequency cut-off (Fc) increases from p6 to p18, thereafter stabilizing out to 8 months at about 6 kHz at room temperature. Assuming that the frequency response is solely limited by the kinetics of prestin (however, see below) and given a Q_10_ of 2^38^, the Fc would approach 15 kHz. The data indicate that the Fc is lower pass in nature than the full auditory capabilities of the mouse, since prestin’s intrinsic kinetics is likely place independent. That is, prestin’s molecular structure, molecular interactions, and especially molecular behavior have yet to show significant differences among OHCs from various turns of the cochlea^39,40^.

Importantly, most developmental prestin characteristics measurable within the *in situ* organ of Corti correspond to those measures obtained in isolated cells^10,21^, i.e., at low measurement frequencies. However, we find that V_h_ in young animals differs from isolated cell studies, and this may be due, for example, to changes in turgor pressure or membrane tension upon isolation. It is notable that for a simple two-state voltage-dependent protein, V_h_ is determined by the ratio of forward and backward transition rates of conformational change. Thus, V_h_ alterations may inform on changes in prestin kinetics. Considering the differences of V_h_ *in situ* vs. in isolated cells, it may be desirable to analyze the effects of prestin mutations, e.g. the “499” knock-in mutation^7^ in the explant, rather than isolated cells.

We find that C_lin_ decreases following the attainment of mature NLC. The decrease corresponds to a decrease in Q_max_, indicating that the absolute number of prestin motors in OHCs decreases with age. This result implies that the surface area of the cells decrease because prestin occupies less surface area, and this is expected since in the prestin KO OHC surface area/length is markedly reduced^41^. The reduction of surface area and prestin in OHCs may have influential effects on the mechanical properties of the cochlear partition. Indeed, in the prestin KO mouse, Mellado Lagarde *et al*. have found that frequency tuning of the partition changes^42^. Thus, we might expect altered hearing in the aging mouse based on the reduction of prestin content that we find.

Why does the frequency response of prestin change during aging? Several possibilities exist. Prestin function itself could change during maturation due to changes in 1) phosphorylation or glycosylation^43,44^; 2) multimerization^45–47^; or 3) molecular crowding within the lateral membrane. Extrinsic influences may also change; for example, during the development of the spaces of Nuel supporting cell rearrangements may occur, and less cell-cell contact could reduce interactive mechanical constraints. Certainly, such types of interactions can affect auditory performance^48^. Finally, cytoskeletal influences on prestin^49,50^ may change during development. We recently, determined that MAP1S, a small microtubule binding protein interacts with both actin and prestin, and co-expression in heterologous cells promotes targeting and alteration in prestin characteristics^51^. Cytoskeletal forces acting on prestin may work to influence conformational switching by the well-known piezoelectric nature of the protein^27,28,30^, which has been extensively modelled^52–54^. Thus, there may well be mechanical filtering of prestin performance depending on whole-cell mechanics that simultaneously impacts the Fc of both NLC and eM, as the two are well coupled^55,56^. In any case, whatever mechanisms that enhance prestin’s frequency response during the developmental likely limits its frequency response in adults. Interestingly, quite low pass estimates (Fc ~1.5 kHz) of adult OHC eM frequency response have been found in the high frequency region of the living gerbil using optical coherence microscopy (OCT) vibrometry^57^. Thus, at this point, the mechanism whereby OHC eM influences very high frequency hearing is under question.
