# Author Correction: Prestin kinetics and corresponding frequency dependence augment during early development of the outer hair cell within the mouse organ of Corti

**DOI:** 10.1038/s41598-020-70538-5

**Published:** 2020-08-27

**Authors:** Jun-Ping Bai, Dhasakumar Navaratnam, Joseph Santos-Sacchi

**Affiliations:** 1 Department of Surgery (Otolaryngology), Yale University School of Medicine, 333 Cedar St, New Haven, CT USA; 2 Department of Cellular and Molecular Physiology, Yale University School of Medicine, 333 Cedar St, New Haven, CT USA; 3 Department of Neuroscience, Yale University School of Medicine, 333 Cedar St, New Haven, CT USA; 4 Department of Neurology, Yale University School of Medicine, 333 Cedar St, New Haven, CT USA

Correction to: *Scientific Reports* https://doi.org/10.1038/s41598-019-52965-1, published online 11 November 2019

This Article contains an error in Figure 5C where the SEM error bars are reported incorrectly.

The correct Figure 5 appears below as Figure 1.

**Figure 1:**
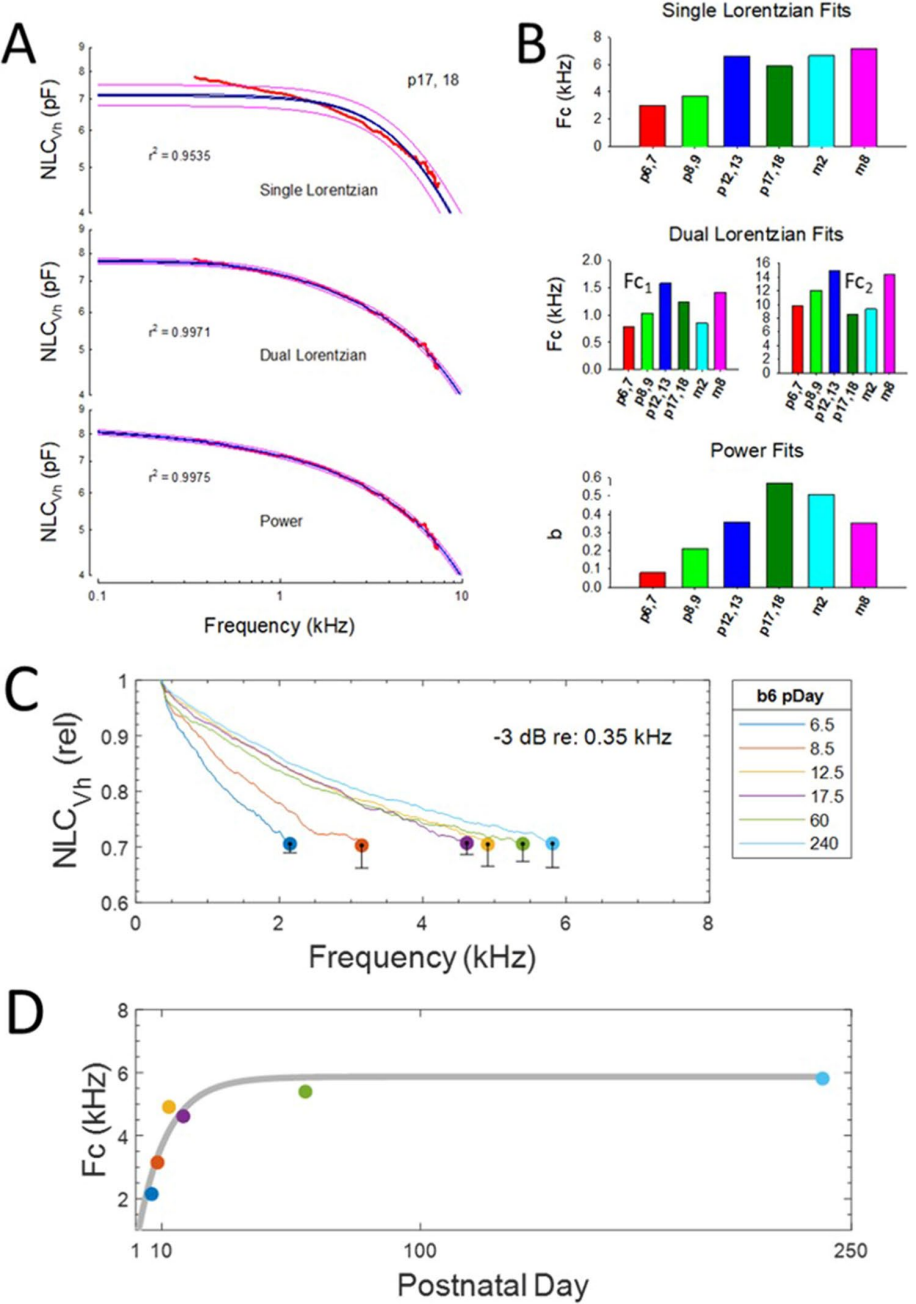
Changes in frequency response of NLC during aging. (**A**) Three types of fits to mean NLC_Vh_ were performed to estimate frequency roll-off. Examples of the 3 fits for p17/18 OHCs are shown. Blue line is fit and red lines are 95% confidence predictions of the fits (done in Sigmaplot). The poorest fit is with a single Lorentzian, followed by dual Lorentzian and power fits (*f* in kHz). Nevertheless, each provides evidence for increases in frequency responsiveness during development. The <a> parameter, as all others, was not constrained, but was not age –dependent and similar for all fits [p6,7 - m8: − 1.4055, − 1.3548, − 1.5980, − 1.1960, − 1.1552, − 1.5031; mean + / − se − 1.369 (0.07)]. The small se indicates little variability. (**B**) Bar plots of frequency cut-off parameters of the fits. (**C**) Another metric of frequency response roll-off was to determine the − 3dB magnitude of NLC_Vh_ relative to 350 Hz values, denoted here with circles. It should be noted that the Fc’s simply reflect the relative roll-off during aging, and their absolute values will differ depending on the reference frequency. During the life span, Fc increases. The se indicates the variability at the cut-off frequencies. (**D**) The Fc data were fit to a power law function in Matlab (grey line; Fc = a.*(1-b.^pDay), where a = 5.867 and b = 0.9065; R^2^ = 0.849), and indicates a stabilization near 6 kHz. P6,7 n = 7; p8,9 n = 7; p12,13 n = 6; p17,18 n = 8; 2 month n = 8; 8 month n = 9.

